# Genetic variants in SERPINA4 and SERPINA5, but not BCL2 and SIK3 are associated with acute kidney injury in critically ill patients with septic shock

**DOI:** 10.1186/s13054-017-1631-3

**Published:** 2017-03-08

**Authors:** Laura M. Vilander, Mari A. Kaunisto, Suvi T. Vaara, Ville Pettilä, the FINNAKI study group, Raili Laru-Sompa, Anni Pulkkinen, Minna Saarelainen, Mikko Reilama, Sinikka Tolmunen, Ulla Rantalainen, Marja Miettinen, Markku Suvela, Katrine Pesola, Pekka Saastamoinen, Sirpa Kauppinen, Ville Pettilä, Kirsi-Maija Kaukonen, Anna-Maija Korhonen, Sara Nisula, Suvi Vaara, Raili Suojaranta-Ylinen, Leena Mildh, Mikko Haapio, Laura Nurminen, Sari Sutinen, Leena Pettilä, Helinä Laitinen, Heidi Syrjä, Kirsi Henttonen, Elina Lappi, Hillevi Boman, Tero Varpula, Päivi Porkka, Mirka Sivula, Mira Rahkonen, Anne Tsurkka, Taina Nieminen, Niina Prittinen, Ari Alaspää, Ville Salanto, Hanna Juntunen, Teija Sanisalo, Ilkka Parviainen, Ari Uusaro, Esko Ruokonen, Stepani Bendel, Niina Rissanen, Maarit Lång, Sari Rahikainen, Saija Rissanen, Merja Ahonen, Elina Halonen, Eija Vaskelainen, Meri Poukkanen, Esa Lintula, Sirpa Suominen, Jorma Heikkinen, Timo Lavander, Kirsi Heinonen, Anne-Mari Juopperi, Tadeusz Kaminski, Fiia Gäddnäs, Tuija Kuusela, Jane Roiko, Sari Karlsson, Matti Reinikainen, Tero Surakka, Helena Jyrkönen, Tanja Eiserbeck, Jaana Kallinen, Vesa Lund, Päivi Tuominen, Pauliina Perkola, Riikka Tuominen, Marika Hietaranta, Satu Johansson, Seppo Hovilehto, Anne Kirsi, Pekka Tiainen, Tuija Myllärinen, Pirjo Leino, Anne Toropainen, Anne Kuitunen, Ilona Leppänen, Markus Levoranta, Sanna Hoppu, Jukka Sauranen, Jyrki Tenhunen, Atte Kukkurainen, Samuli Kortelainen, Simo Varila, Outi Inkinen, Niina Koivuviita, Jutta Kotamäki, Anu Laine, Tero Ala-Kokko, Jouko Laurila, Sinikka Sälkiö, Simo-Pekka Koivisto, Raku Hautamäki, Maria Skinnar

**Affiliations:** 1 Division of Intensive Care Medicine, Department of Anesthesiology, Intensive Care and Pain Medicine, University of Helsinki and Helsinki University Hospital, Helsinki, Finland; 2 Institute for Molecular Medicine Finland (FIMM), University of Helsinki, Helsinki, Finland; 3 Inselspital, Bern University Hospital, University of Bern, Bern, Switzerland

**Keywords:** Acute kidney injury, Genetic susceptibility, Sepsis, Septic shock, Apoptosis, *BCL2*, *SERPINA4*, *SERPINA5*, *SIK3*

## Abstract

**Background:**

Acute kidney injury (AKI) is a multifactorial syndrome, but knowledge about its pathophysiology and possible genetic background is limited. Recently the first hypothesis-free genetic association studies have been published to explore individual susceptibility to AKI. We aimed to replicate the previously identified associations between five candidate single nucleotide polymorphisms (SNP) in apoptosis-related genes *BCL2, SERPINA4, SERPINA5*, and *SIK3* and the development of AKI, using a prospective cohort of critically ill patients with sepsis/septic shock, in Finland.

**Methods:**

This is a prospective, observational multicenter study. Of 2567 patients without chronic kidney disease and with genetic samples included in the Finnish Acute Kidney Injury (FINNAKI) study, 837 patients had sepsis and 627 patients had septic shock. AKI was defined according to the Kidney Disease: Improving Global Outcomes (KDIGO) criteria, considering stages 2 and 3 affected (severe AKI), stage 0 unaffected, and stage 1 indecisive. Genotyping was done using iPLEX^TM^ Assay (Agena Bioscience). The genotyped SNPs were rs8094315 and rs12457893 in the intron of the *BCL2* gene, rs2093266 in the *SERPINA4* gene, rs1955656 in the *SERPINA5* gene and rs625145 in the *SIK3* gene. Association analyses were performed using logistic regression with PLINK software.

**Results:**

We found no significant associations between the SNPs and severe AKI in patients with sepsis/septic shock, even after adjustment for confounders. Among patients with septic shock (252 with severe AKI and 226 without AKI (149 with KDIGO stage 1 excluded)), the SNPs rs2093266 and rs1955656 were significantly (odds ratio 0.63, *p* = 0.04276) associated with stage 2–3 AKI after adjusting for clinical and demographic variables.

**Conclusions:**

The SNPs rs2093266 in the *SERPINA4* and rs1955656 in the *SERPINA5* were associated with the development of severe AKI (KDIGO stage 2–3) in critically ill patients with septic shock. For the other SNPs, we did not confirm the previously reported associations.

**Electronic supplementary material:**

The online version of this article (doi:10.1186/s13054-017-1631-3) contains supplementary material, which is available to authorized users.

## Background

In critically ill patients, the incidence of acute kidney injury (AKI) is high - 39% in the recent prospective, observational, multicenter study, the Finnish Acute Kidney Injury (FINNAKI) study, which was conducted in Finnish intensive care units (ICUs) [1]. In other prospective studies in critically ill patients the incidence of AKI is reported as 24–66% [2, 3]. Although the pathophysiology of AKI has been investigated, there is no single explanation for the condition due to the multifactorial nature of the syndrome. Sepsis is the most common factor predisposing to AKI in the critically ill. In patients with sepsis the incidence of AKI is 31–53% [4–6] and in patients with septic shock it is even higher, at 47–61% [7, 8]. The previously identified risk factors for septic AKI, such as age, sex, and baseline comorbidities, have failed to reliably predict individual risk of septic AKI [9]. Thus, it is plausible that genetic variability between individuals may explain a significant part of the risk.

Genetic predisposition to AKI has been previously studied in candidate genes, by testing associations between single nucleotide polymorphisms (SNP) from candidate genes and a phenotype. Our recent systematic review confirmed that despite some positive associations there are no conclusive data [10]. A study by Frank et al*.* [11] provided one of the very first hypothesis-free approaches to septic AKI; it comprised 887 patients in septic shock defined according to American College of Chest Physicians/Society of Critical Care Medicine (ACCP/SCCM) criteria [12], who were genotyped using a large-scale genotyping microarray. In this study, five SNPs were associated with AKI after validating the results in an additional sample. Interestingly, the associated SNPs are in the apoptosis pathway genes. The SNP rs8094315 and SNP rs12457893 are located in the intron of *B-cell CLL/lymphoma 2 (BCL2)* –gene. The SNPs rs2093266 in the *serpin peptidase inhibitor, clade A (alpha-1 antiproteinase, antitrypsin), member 4 (SERPINA4)* gene and SNP rs1955656 in the *serpin peptidase inhibitor, clade A (alpha-1 antiproteinase, antitrypsin), member 5 (SERPINA5)* gene are in complete linkage disequilibrium (LD). There was also association between the SNP rs625145 in the *salt-inducible kinase family 3 (SIK3)* gene and AKI. In this study we aimed to replicate the aforementioned findings [9] in a prospective cohort of critically ill patients with sepsis.

## Methods

### Study population

This study is a predetermined genetic study of the prospective, observational, multicenter FINNAKI study, in which patients were recruited from 17 Finnish ICUs. Details of the FINNAKI study have been published elsewhere [1] and are presented in Additional file 1. The Ethics Committee of the Department of Surgery in Helsinki University Hospital approved the study. An informed separate written consent for genetic samples was obtained from the patient or next of kin at the initiation, with the option of deferred consent. The main study ended on 1 February 2012, and recruitment was extended until 30 April 2012 to achieve an adequate number of patients with sepsis. The study was conducted according to the Declaration of Helsinki.

### Data collection

We collected the study-specific data items (approximately 80% of the data) on admission and daily until day 5 or ICU discharge using an electronic case report form (CRF). Data comprised previous and current health status, medication, risk factors for AKI, laboratory values and operations preceding ICU admission, sepsis and related organ dysfunctions, and focus of infection. Presence of AKI and/or sepsis was screened until ICU discharge or day 5 at the latest if the patient was still in the ICU. Routine data were collected with the help of the Finnish Intensive Care Consortium, which consists of 25 ICUs nationwide.

### Definitions

For staging of AKI, plasma creatinine was measured daily and urine output hourly. We defined and staged AKI according to the new Kidney Disease: Improving Global Outcomes (KDIGO) AKI criteria [13]. We sought to compare patients with a severe phenotype of AKI (KDIGO stage 2–3) to patients with no AKI. Thus, we excluded patients with KDIGO stage-1 AKI from the current analysis because their phenotype may be seen as indecisive. We defined sepsis and septic shock according to the ACCP/SCCM definitions [12], a definition also used in the previous report [11].

### Blood sampling and DNA extraction

Whole blood was collected at enrollment and after separation of plasma, stored at -80 °C for subsequent DNA extraction. DNA was isolated on a Chemagic 360 instrument (Perkin Elmer, Baesweiler, Germany), based on magnetic bead technology, using a Chemagic DNA Blood10k Kit according to the manufacturer’s instructions. DNA concentrations were determined using UV light and PicoGreen methods, and samples were diluted into 10 ng/μl for genotyping.

### Genotype analysis

Genotyping was performed at the Technology Centre of the Institute for Molecular Medicine Finland (FIMM), University of Helsinki. The genotyping was done using Agena MassARRAY® system and the iPLEX^TM^ Gold Assay (Agena Bioscience^TM^, San Diego, CA, USA). This method has excellent success (>95%) and accuracy (100%) [14]. Genotyping reactions were performed on 20 ng of dried genomic DNA in 384-well plates according to the manufacturer’s recommendations and using their reagents [15]. Both polymerase chain reaction (PCR) and extension primers were designed using MassARRAY Assay Design software (Agena Bioscience^TM^) (Additional file 2). The data were collected using the MassARRAY Compact System (Agena Bioscience^TM^) and the genotypes were called using TyperAnalyzer software (Agena Bioscience^TM^).

We examined genotyping quality by a detailed quality control procedure consisting of success rate check, duplicated samples, water controls, and Hardy-Weinberg equilibrium (HWE) testing. In addition, the genotype calls were checked manually and corrected when necessary. Genotyping personnel were blinded to the clinical status of the patients.

### Statistical analysis

We compared the clinical and demographic variables to test for significant differences between groups using the Fisher exact test for categorical variables and the Mann-Whitney *U* test for continuous variables. We present data as medians and interquartile ranges, or absolute values and percentages. Statistical analyses of the demographic and clinical variables were performed using the SPSS Statistics version 22 (IBM Corp., Armonk, NY, USA).

Associations between AKI and SNP were adjusted for clinical characteristics that differed significantly between patients with and without AKI. In addition to the univariate analysis we performed multivariate analysis using logistic regression by entering variables, selecting characteristics with a *p* value <0.2 in univariate analysis. The percentage of missing data was 15.9%. The missing data were imputed on the assumption of being unaffected for categorical variables and on the median for continuous variables.

Genetic association tests were performed using logistic regression and the PLINK software [16]. As in the previous study an additive genetic model was assumed. However, confirmatory tests were performed using recessive and dominant genetic models. In the primary analysis patients with sepsis and AKI (KDIGO stage 2–3) were compared to patients with sepsis but no AKI. Secondary analyses were performed including cases and controls from the entire cohort, and was performed separately only in those with septic shock. As this was a replication study, we considered all *p* values <0.05 significant.

### Power calculations

Power calculations were made using Genetic Power Calculator [17], assuming allele frequencies and odds ratios from the validation cohort from the study by Frank et al*.* [11], and with the assumption of 299 patients and 354 controls, a prevalence of AKI of 39,% and a type I error rate of 0.005. For detailed power calculations for each SNP see Additional file 3.

## Results

### Patients

**Fig. 1:**
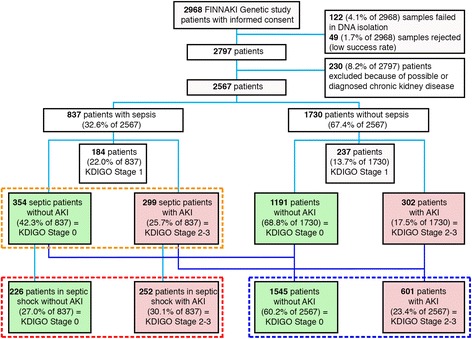
Study flowchart. *AKI* acute kidney injury, *KDIGO* Kidney Disease: Improving Global Outcomes, *FINNAKI* Finnish Acute Kidney Injury study

We prospectively enrolled 2968 ICU patients in the FINNAKI genetic study, as presented in the study flowchart (Fig. 1). After excluding ineligible patients, there were 2567 critically ill patients, of whom 837 (32.6%) had sepsis. Of these, 299 (35.7% of the 837) patients developed KDIGO stage 2–3 AKI and 354 (42.3% of 837) patients with no AKI served as controls (Fig. 1, orange dashed line). Thus, the genetic associations were studied among 653 patients with sepsis, of whom 478 (73.2%) had septic shock. Among patients with septic shock, 252 (40.2% of 627) had severe AKI and 226 (36.0% of 627) did not have AKI (Fig. 1, red dashed line). In the entire genetic cohort, there were 601 (23.4% of 2567) patients who developed KDIGO stage 2–3 AKI and 1545 (60.2% of 2567) patients who did not develop AKI (Fig. 1, blue dashed line).

The baseline characteristics of the patients with sepsis who did and did not have AKI are presented in Table 1. The baseline characteristics of the patients with septic shock who did and did not have AKI are presented in Table 2. The baseline characteristics of the entire cohort are presented in Additional file 4.

**Table 1:** Demographic and baseline characteristics of the patients with sepsis according to the presence of acute kidney injury

Characteristics	Patients with data available, *n*	KDIGO 0 (n = 354)	KDIGO 2–3 (n = 299)	All patients (n = 653)	*P* value
Age (years)	653	63 (52–73)	65 (54.5–75)	63 (53–74)	0.046
Gender (male)	653	234 (66.1%)	183 (61.2%)	417 (63.9%)	0.195
BMI (kg/m^2^)	651	26.0 (23.1–29.2)	27.3 (24.5–30.8)	26.5 (23.5–29.6)	0.001
Arterial hypertension	651	164 (46.6%)	159 (53.2%)	323 (49.6%)	0.099
Diabetes	653	68 (19.2%)	82 (27.4%)	150 (23.0%)	0.015
Arteriosclerosis	648	36 (10.3%)	42 (14.1%)	78 (12.0%)	0.147
COPD	649	45 (12.8%)	19 (6.4%)	64 (9.9%)	0.008
Chronic liver disease	647	17 (4.9%)	24 (8.1%)	41 (6.3%)	0.106
Systolic heart failure	649	39 (11.1%)	24 (8.1%)	63 (9.7%)	0.231
Thromboembolus	649	26 (7.4%)	17 (5.7%)	43 (6.6%)	0.432
Rheumatic disease	648	26 (7.4%)	18 (6.1%)	44 (6.8%)	0.534
Serum creatinine					
Baseline (μmol/L)	653	80.3 (68.0–94.0)	77.0 (66.5–93.0)	79.0 (67.0–93.2)	0.318
Maximum (μmol/L)	604	72.0 (55.0–89.0)	227.0 (166.0–321.0)	106.5 (68.5–221.5)	<0.001
Pre-ICU daily medication					
ACE inhibitor or ARB	640	113 (32.6%)	115 (39.2%)	228 (35.6%)	0.082
NSAID	624	33 (9.7%)	42 (14.7%)	75 (12.0%)	0.064
Aspirin	645	81 (23.1%)	73 (24.7%)	154 (23.9%)	0.644
Diuretic	643	93 (26.8%)	88 (29.7%)	181 (28.1)	0.429
Metformin	647	48 (13.7%)	47 (15.8%)	95 (14.7%)	0.504
Statin	644	86 (24.6%)	79 (26.8%)	165 (25.6%)	0.587
Immunosuppressives	646	29 (8.3%)	28 (9.5%)	57 (8.8%)	0.581
Corticosteroids	649	41 (11.6%)	32 (10.8%)	73 (11.2%)	0.803
Warfarin	647	39 (11.1%)	41 (13.8%)	80 (12.4%)	0.338
Treatments administered 48 h before admission					
Contrast medium	652	83 (23.5%)	51 (17.1%)	134 (20.6%)	0.052
Aminoglycoside antibiotics	653	4 (1.1%)	5 (1.7%)	9 (1.4%)	0.739
Peptidoglycan antibiotics	653	17 (4.8%)	11 (3.7%)	28 (4.3%)	0.563
ACE inhibitor or ARB	641	77 (22.1%)	72 (24.7%)	149 (23.2%)	0.454
NSAID	615	52 (15.6%)	40 (14.2%)	92 (15.0%)	0.651
Amfoterisin B	653	1 (0.3%)	2 (0.7%)	3 (0.5%)	0.596
Diuretics	637	119 (34.6%)	117 (39.9%)	236 (37.0%)	0.188
Colloids (gelatin or starch)	620	102 (30.8%)	117 (40.5%)	219 (35.3%)	0.015
Albumin	647	4 (1.1%)	6 (2.0%)	10 (1.5%)	0.526
Emergency admission	648	343 (98.0%)	292 (98.0%)	635 (98.0%)	1.0
Operative admission	652	88 (24.9%)	66 (22.1%)	154 (23.6%)	0.459
SAPS II score 24 h without renal and age components	649	24.0 (17.0–30.0)	26.0 (20.0–37.0)	25.0 (18.0–33.0)	<0.001
Mechanical ventilation	653	234 (66.1%)	221 (73.9%)	455 (69.7%)	0.033
White blood cell count, maximum (10^9^/L)	583	11.8 (7.9–16.5)	12.2 (7.6–17.8)	11.9 (7.7–17.2)	0.469
Platelet count, minimum (10^9^/L)	632	199.5 (141.0–271.0)	184.0 (112.0–259.0)	191.5 (128.5–265.0)	0.028
Source of infection	653				<0.001
Lung		181 (51.1%)	100 (33.4%)	281 (43.0%)	
Abdomen		62 (17.5%)	78 (26.1%)	140 (21.4%)	
Urinary tract		10 (2.8%)	26 (8.7%)	36 (5.5%)	
Skin		25 (7.1%)	26 (8.7%)	51 (7.8%)	
Others		19 (5.4%)	9 (3.0%)	28 (4.3%)	
Multiple sources		21 (5.9%)	16 (5.4%)	37 (5.7%)	
Unknown source of infection		36 (10.2%)	44 (14.7%)	80 (12.3%)	

Results presented as median (interquartile range) for continuous variables and total number (percent of affected in a group) for categorical variables. Continuous variables analyzed by the independent samples Mann-Whitney *U* test and categorical variables by Fisher’s exact test. *KDIGO* Kidney Disease: Improving Global Outcomes, *BMI* body mass index, *COPD* chronic obstructive pulmonary disease, *AR* *B* angiotensin receptor blocker, *NSAID* non-steroidal anti-inflammatory drug, *ACE* angiotensin-converting enzyme, *SAPS* simplified acute physiology score

**Table 2:** Demographic and baseline characteristics of patients with septic shock according to the presence of acute kidney injury

Characteristics	Patients with data available, *n*	KDIGO 0 (n = 226)	KDIGO 2–3 (n = 252)	All patients (n = 478)	*P* value
Age (years)	478	63.0 (52.0–73.0)	64.5 (54.0–75.0)	63.5 (53.0–74.0)	0.138
Gender (male)	478	155 (68.6%)	156 (61.9%)	311 (65.1%)	0.149
BMI (kg/m^2^)	476	26.0 (23.0–29.0)	27.3 (24.5–30.5)	26.5 (23.5–29.5)	0.001
Arterial hypertension	476	104 (46.4%)	137 (54.4%)	241 (50.6%)	0.098
Diabetes	478	42 (18.6%)	65 (25.8%)	107 (22.4%)	0.062
Arteriosclerosis	475	21 (9.4%)	36 (14.3%)	57 (12.0%)	0.120
COPD	474	28 (12.5%)	19 (7.6%)	47 (9.9%)	0.090
Chronic liver disease	473	10 (4.5%)	22 (8.8%)	32 (6.8%)	0.069
Systolic heart failure	475	22 (9.8%)	22 (8.8%)	44 (9.3%)	0.752
Thromboembolus	475	14 (6.2%)	14 (5.6%)	28 (5.9%)	0.846
Rheumatic diseases	475	19 (8.5%)	17 (6.8%)	36 (7.6%)	0.493
Serum creatinine					
Baseline (μmol/L)	478	82.5 (69.0–93.4)	77.0 (67.0–93.0)	79.7 (67.0–93.0)	0.236
Maximum (μmol/L)	478	69.0 (50.0–89.0)	226.5 (166.0–323.0)	113.5 (67.0–230.0)	<0.0001
Pre-ICU daily medication					
ACE inhibitor or ARB	465	78 (35.6%)	96 (39.0%)	174 (37.4%)	0.502
NSAID	453	21 (9.8%)	35 (14.6%)	56 (12.4%)	0.152
Aspirin	471	52 (23.4%)	59 (23.7%)	111 (23.6%)	1.000
Diuretic	468	57 (26.0%)	76 (30.5%)	133 (28.4%)	0.305
Metformin	473	30 (13.5%)	38 (15.2%)	68 (14.4%)	0.602
Statin	469	55 (24.9%)	61 (24.6%)	116 (24.7%)	1.000
Immunosuppressives	471	19 (8.5%)	26 (10.5%)	45 (9.6%)	0.531
Corticosteroids	474	26 (11.6%)	29 (11.6%	55 (11.6%)	1.000
Warfarin	472	26 (11.7%)	34 (13.6%)	60 (12.7%)	0.581
Treatments administered 48 h before admission					
Contrast medium	477	58 (25.8%)	46 (18.3%)	104 (21.8%)	0.059
Aminoglycoside antibiotics	478	2 (0.9%)	5 (2.0%	7 (1.5%)	0.455
Peptidoglycan antibiotics	478	12 (5.3%)	10 (4.0%)	22 (4.6%)	0.519
ACE inhibitor or ARB	468	43 (19.3%)	62 (25.3%)	105 (22.4%)	0.122
NSAID	448	28 (13.4%)	36 (15.1%)	64 (14.3%)	0.685
Amfoterisin B	478	0 (0.0%)	2 (0.8%)	2 (0.4%)	0.500
Diuretics	464	70 (32.1%)	97 (39.4%)	167 (36.0%)	0.121
Colloids (gelatin or starch)	452	76 (36.4%)	105 (43.2%)	181 (40.0%)	0.149
Albumin	474	4 (1.8%)	6 (2.4%)	10 (2.1%)	0.756
Emergency admission	474	217 (97.3%)	245 (97.6%)	462 (97.5%)	1.000
Operative admission	477	69 (30.5%)	58 (23.1%)	127 (26.6%)	0.078
SAPS II score 24 h without renal and age components	475	25.0 (19.0–33.0)	26.0 (21.0–37.0)	26.0 (20.0–35.0)	0.054
Mechanical ventilation	478	177 (78.3%)	197 (78.2%)	374 (78.2%)	1.000
White blood cell count, maximum (10^9^/L)	428	12.1 (8.7–16.8)	12.8 (7.6–18.7)	12.3 (8.1–17.4)	0.794
Platelet count, minimum (10^9^/L)	458	191.0 (140.0–260.0)	177.0 (108.5–259.0)	185.0 (121.0–259.0)	0.065
Source of infection	478				0.001
Lung		114 (50.4%)	83 (32.9%)	197 (41.2%)	
Abdomen		46 (20.4%)	68 (27.0%)	114 (23.8%)	
Urinary tract		7 (3.1%)	20 (7.9%)	27 (5.6%)	
Skin		16 (7.1%)	22 (8.7%)	38 (7.9%)	
Others		11 (4.9)	8 (3.2%)	19 (4.0%)	
Multiple sources		13 (5.8%)	14 (5.6%)	27 (5.6%)	
Unknown source of infection		19 (8.4%)	37 (14.7%)	56 (11.7%)	

Results presented as median (interquartile range) for continuous variables and total number (percent of affected in a group) for categorical variables. Continuous variables analyzed by the independent samples Mann-Whitney *U* test and categorical variables by Fisher’s exact test. *KDIGO* Kidney Disease: Improving Global Outcomes, *BMI* body mass index, *COPD* chronic obstructive pulmonary disease, *AR* *B* angiotensin receptor blocker, *NSAID* non-steroidal anti-inflammatory drug, *ACE* angiotensin-converting enzyme, *SAPS* simplified acute physiology score

### Genetic associations

All of the polymorphisms tested were in HWE. None of the five SNPs investigated, rs8094315 (odds ratio (OR) 1.10, *p* = 0.48), rs12457893 (OR 1.02, *p* = 0.87), rs2093266 (OR 0.77, *p* = 0.15), rs1955656 (OR 0.77, *p* = 0.15), and rs625145 (OR 0.88, *p* = 0.38), was significantly associated with AKI in our analysis in patients with sepsis in the additive genetic model (Table 3). The SNPs rs2093266 and rs1955656 are in complete linkage disequilibrium (LD) and thus present identical results.

**Table 3:** Association between acute kidney injury and the polymorphisms studied in 653 patients with sepsis (additive genetic model)

					Univariate		Multivariate	
SNP	Chr	Base-pair position	Gene and alleles (major/minor)	Minor allele frequency^a^	Odds ratio (95% CI)	*P* value	Odds ratio (95% CI)^b^	*P* value
rs625145	11	116857220	*SIK3* A/T	0.20/0.21	0.88 (0.67–1.17)	0.38	0.93 (0.68–1.25)	0.62
rs1955656	14	94579038	*SERPINA5* G/A	0.10/0.12	0.77 (0.53–1.10)	0.15	0.75 (0.51–1.10)	0.14
rs2093266	14	94566450	*SERPINA4* G/A	0.10/0.12	0.77 (0.53–1.10)	0.15	0.75 (0.51–1.10)	0.14
rs8094315	18	63268814	*BCL2* A/G	0.24/0.22	1.10 (0.85–1.43)	0.48	1.08 (0.81–1.43)	0.61
rs12457893	18	63258928	*BCL2* A/C	0.37/0.37	1.02 (0.81–1.29)	0.87	1.06 (0.82–1.36)	0.67

^a^Patients/controls. ^b^Adjusted for age, body mass index, diabetes mellitus, mechanical ventilation, minimum platelet count, use of non-steroidal anti-inflammatory drugs as daily medication, chronic pulmonary obstructive disease, administration of contrast medium prior to ICU admission, administration of colloids prior to ICU admission, simplified acute physiology score II without age or renal components, operative admission, and source of infection. *SNP* single nucleotide polymorphism, *Chr* chromosome

In logistic regression analysis in patients with sepsis, higher body mass index (BMI), not having chronic obstructive pulmonary disease (COPD), use of non-steroidal anti-inflammatory drugs (NSAID) as daily medication, administration of contrast medium prior to ICU admission, simplified acute physiology score II (SAPS II) without renal or age components, and source of infection were significantly associated with KDIGO stage 2–3 AKI (Additional file 5). Adjustment for these clinical and demographic factors in the patients with sepsis did not change the results for genetic association, which were not statistically significant (Table 3).

**Fig. 2:**
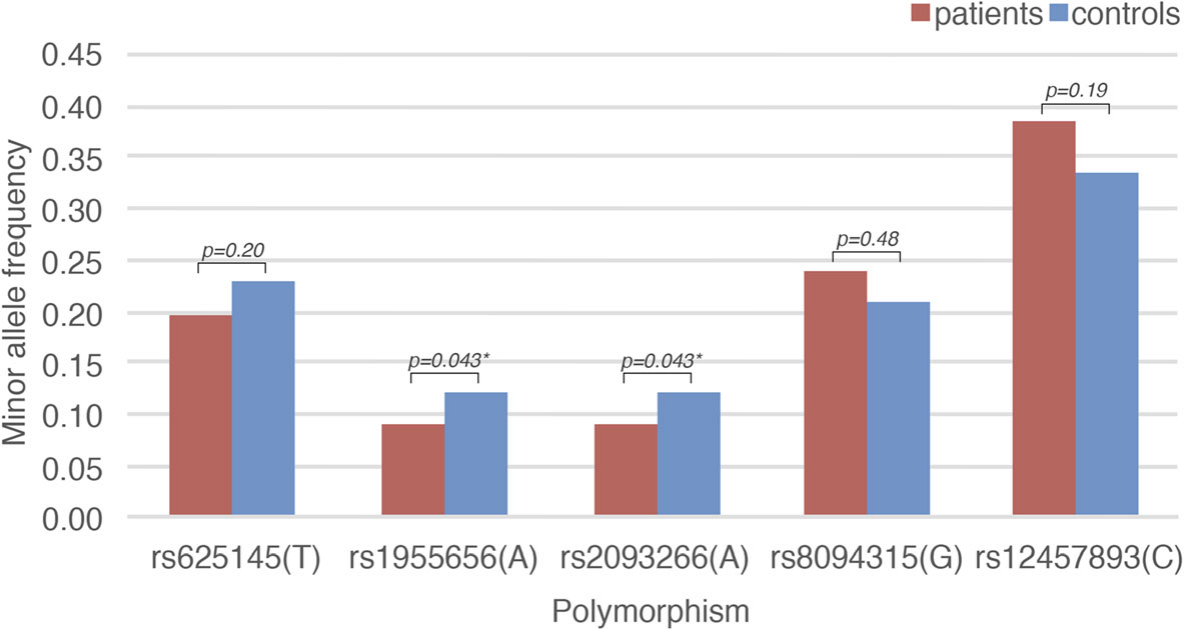
Minor allele frequencies of all polymorphisms studied in patients with septic shock (n = 478). Patients (*red*) had severe acute kidney injury (AKI) (Kidney Disease: Improving Global Outcomes (KDIGO) stage 2 or 3 AKI, n = 252) and controls (*blue*) were ICU patients without AKI. The *p* values shown above the *bars* are for the multivariate tests of association between the polymorphisms and AKI. *Significant *p* values

In patients with septic shock, none of the investigated SNPs was significantly associated with AKI in univariate analysis (Table 4). After adjusting for clinical and demographic variables that remained significant in logistic regression (BMI, use of NSAID as daily medication, arteriosclerosis, COPD, administration of contrast medium prior to ICU admission, administration of colloids prior to ICU admission, SAPS II without age or renal components, operative admission, and source of infection (Additional file 6)) SNPs rs2093266 and rs1955656 were significantly associated with AKI (OR 0.63, 95% CI 0.40 to 0.98, *p* = 0.043, for each) (Table 4, Fig. 2). The carriers of the minor alleles of these SNPs (A and A, respectively) had a decreased risk of developing AKI. This association was in the same direction as in the previous report. The minor allele frequencies of all investigated SNPs in patients with septic shock are presented in Fig. 2.

**Table 4:** Association between acute kidney injury and the polymorphisms studied in 478 patients in septic shock (additive genetic model)

Single nucleotide polymorphism	Univariate odds ratio (95% confidence interval)	Univariate *P* value	Multivariate odds ratio (95% confidence interval)^a^	Multivariate *P* value
rs625145	0.81 (0.59–1.11)	0.19	0.80 (0.56–1.13)	0.20
rs1955656	0.71 (0.46–1.07)	0.10	0.63 (0.40–0.98)	0.043
rs2093266	0.71 (0.46–1.07)	0.10	0.63 (0.40–0.98)	0.043
rs8094315	1.19 (0.88–1.62)	0.27	1.13 (0.81–1.58)	0.48
rs12457893	1.24 (0.94–1.62)	0.13	1.22 (0.91–1.64)	0.19

^a^Adjusted for body mass index, use of non-steroidal anti-inflammatory drugs as daily medication, arteriosclerosis, chronic obstructive pulmonary disease, administration of contrast medium prior to ICU admission, administration of colloids prior to ICU admission, simplified acute physiology score II without age or renal components, operative admission, and source of infection

When the same analyses were performed in the entire genetic cohort, there was no evidence of association between any of the SNPs and AKI in univariate or multivariate models when adjusting for clinical and demographic variables that remained significant in logistic regression (Additional files 7 and 8).

Using recessive and dominant genetic models did not change the results: SNPs rs8094315, rs12457893 and rs625145 were not associated with AKI in any of the subgroups studied. The protective association with SNPs rs2093266 and rs1955656 prevailed in patients in septic shock in the dominant adjusted model (OR 0.59, *p* = 0.034, for each) (Additional file 9). The self-assessed quality score of the study was 9/10 [18] (Additional file 10).

## Discussion

In this study we confirmed the previously reported association between AKI and two polymorphisms, rs2093266 and rs1955656 in apoptosis-related genes *SERPINA* 4 and *SERPINA5* in patients with septic shock. Both in our study and in the original study the minor alleles of these SNPs provided protection from AKI. The association signal was seen in both additive and dominant genetic models but not in the recessive model, suggesting that merely one protective allele is sufficient.

Our study was based on a previous report suggesting that polymorphisms within apoptosis-related genes may be associated with the development of AKI. In this study comprising 887 patients with septic shock, AKI was associated with five SNPs [11]. To our knowledge this is the first successful replication of two of these SNPs. There was no evidence in our cohort of significant association between AKI and the other three polymorphisms tested, rs8094315, rs12457893, and rs625145.

The SNP rs2093266 is located in an intronic region of *SERPINA4* gene encoding kallistatin, a serine proteinase inhibitor that has multiple regulatory roles in biological processes [19, 20]. In kallistatin there are two functional domains, an active site and a heparin-binding site, through which it regulates several signaling and biological pathways. Along with its functions in relation to apoptosis, it has anti-inflammatory, antioxidant, vasodilator and angiogenesis inhibiting functions [21]. All these mechanisms are relevant in septic AKI and could thus explain the better outcome.

In recent mouse model studies, Kallistatin has been associated with attenuated inflammation and organ injury and decreased mortality in established sepsis [22], and with improved survival in sepsis-related acute lung injury [23]. Of note, it may also have a renoprotective role against diabetic nephropathy [24]. rs1955656 in *SERPINA5* is strongly correlated with rs2093266 and as suspected before, this association with AKI could be driven by rs2093266. Notably, the product of *SERPINA5* is known to inhibit activated protein C. This protein C inhibitor (PCI) plays a role in tumor growth and metastasis through its effect on blood coagulation, but it has also been suggested to inhibit the anti-inflammatory activity of activated protein C [25, 26]. This inflammatory function could possibly explain the protection against septic AKI.

Of the other three SNPs studied, two (rs8094315 and rs12457893) are located in the introns of the apoptosis-related gene *BCL2* encoding an integral outer mitochondrial membrane protein BCL2 that blocks the apoptotic death of certain cells [27]. The SNP rs625145 is in *SIK3*, coding for a member of the AMP-activated protein kinase family that affects the regulation of several genes. This protein is found to suppress inflammatory molecule gene expression in macrophages stimulated with lipopolysaccharides (LPS) [28].

There is no definite consensus on the pathophysiology of AKI, and until recently the most common approach has been to test for association with known genetic variants. These variants have often come up in another phenotype, commonly chronic kidney disease, and, thus, may serve as poor markers of AKI [29, 30]. So far, the most studied polymorphisms are within inflammatory mediator genes [10].

Apoptosis-related genes are good candidates for AKI because there is some evidence that apoptosis is an important mechanism in septic AKI. In a murine model of septic AKI the pathophysiology appears to differ from that of ischemia-reperfusion insult, lacking signs of tubular cell injury by necrosis but showing a substantial number of tubular cells undergoing apoptosis [31]. Lerolle et al*.* found that in post-mortem kidney biopsies of patients who died of septic shock (n = 19) there was a marked increase in apoptosis and capillary leukocytic infiltration in comparison with patients with trauma and ICU controls [32]. However, there were contradicting results in a separate sample of post-mortem kidney biopsies in patients who died of sepsis [33]. The nature of septic AKI alone appears to be diverse, with temporal and individual variation, and the role of apoptosis is inconclusive [34].

We also tested the association between the five SNPs and development of AKI in all patients with sepsis, but the results were negative. The phenotype of septic shock differs from that of sepsis without shock, reflecting a more severe form of illness and a greater risk of death. Thus, we can speculate that the associations between SNPs and AKI detected in these patients, although not generalizable to the septic cohort, can predict better survival for the carriers of the protective allele in terms of AKI.

The strength of our study is that it was a prospective, relatively large, multicenter study comprising consecutive patients. In addition we used the KDIGO criteria to provide a robust phenotype of AKI and critically ill patients without AKI as controls to increase the power of our study.

There are some important limitations in our study. First, 171 genetic samples (5.8%) could not be analyzed due to failure in the DNA isolation phase or to rejection of samples because of a low genotyping success rate. Second, we did not collect data ont ethnicity. However, 99.9% of Finnish-speaking inhabitants are Caucasian. Third, individual genetic susceptibility factors can be expected to increase the AKI risk with values just exceeding an OR of 1 [35]. In our power calculations we used the ORs reported in the original study, and, thus, our study might have been underpowered to replicate the results for *BCL2* and *SIK3*. Fourth, we did not adjust the *p* values for multiple testing, in this replication of positive findings.

Finally, in contrast to the previous study [11] we excluded patients with chronic kidney disease. Furthermore, we aimed to strengthen the phenotype by comparing patients without AKI to patients with more severe AKI (KDIGO stage 2–3), thus excluding those with KDIGO stage 1. We reasoned that this group would include patients with only KDIGO stage-1 urine output (not included in [11]) criteria, whose phenotype clearly differs from that of more severe acute kidney.

These findings provide some interesting questions for future research. The functions of the SNPs rs2093266 in the *SERPINA4* and rs1955656 in the *SERPINA5* for the protein products are yet to be determined. If further studies can provide independent evidence supporting the role of these SNPs in AKI susceptibility, information about the genotype of either of these SNPs may add to the battery of risk-predicting tools. However, the effect size of the SNPs is too small for the genotype information to work as an independent biomarker in a clinical setting.

## Conclusions

In this study we aimed to replicate the previous findings associating polymorphisms within apoptosis-related genes to AKI. We found that SNPs rs2093266 in the *SERPINA4* and rs1955656 in the *SERPINA5* were associated with KDIGO stage 2–3 AKI in critically ill patients in septic shock.

## Additional files

Additional file 1: FINNAKI study enrollment and inclusion of patients. Report of the enrollment period and inclusion and exclusion criteria. (DOC 22 kb)
Additional file 2: Primer sequences. Polymerase chain reaction (PCR) 1 and 2 and extension primer sequences. (DOC 30 kb)
Additional file 3: Detailed power calculations for each SNP, for power to report the additionional risk given known minor allele frequencies and risk ratios. (DOC 22 kb)
Additional file 4: Demographic and baseline characteristics of the entire cohort according to the presence of acute kidney injury: the main demographic and baseline characteristics of the patients included in the FINNAKI genetic study. (DOC 86 kb)
Additional file 5: Logistic regression (the “enter” method) in patients with sepsis (n = 653) in differing demographic variables, missing data imputed. Number (*N*) of imputed values and percentages of the cohort are given. Results of logistic regression in patients with sepsis showing that higher BMI, not having COPD, use of NSAID as daily medication, administration of contrast medium prior to ICU admission, SAPS II score without renal or age components, and source of infection were significantly associated with KDIGO stage 2–3 AKI. (DOC 54 kb)
Additional file 6: Logistic regression (“enter” method) in patients with septic shock (n = 478) in differing demographic variables, missing data imputed. Number (*N*) of imputed values and percentages of the cohort are given. Results of logistic regression in patients with septic shock showing that BMI, use of NSAID as daily medication, arteriosclerosis, COPD, administration of contrast medium prior to ICU admission, administration of colloids prior to ICU admission, SAPS II without age or renal components, operative admission, and source of infection were significantly associated with KDIGO stage 2–3 AKI. (DOC 53 kb)
Additional file 7: Association between acute kidney injury and the polymorphisms studied in all genotyped patients (n = 2146) (additive genetic model). Association between acute kidney injury and the studied polymorphisms in all genotyped patients was tested in univariate and multivariate models. No significant associations are reported. (DOC 35 kb)
Additional file 8: Logistic regression (“enter” method) in all genotyped patients (n = 2146) in differing demographic variables, missing data imputed. Number (*N*) of imputed values and the percentages of the cohort are given. Logistic regression in all genotyped patients showed that BMI, chronic liver disease, use of NSAID as daily medication, use of warfarin as daily medication, administration of contrast medium prior to ICU admission, administration of colloids prior to ICU admission, administration of albumin prior to ICU admission, maximum white blood cell count, minimum platelet count, SAPS II without age or renal components, operative admission, and source of infection were significantly associated with KDIGO stage 2–3 AKI. (DOC 72 kb)
Additional file 9: Association between acute kidney injury and the studied polymorphisms, recessive and dominant genetic models. Association between acute kidney injury and the polymorphisms studied in septic patients, patients with septic shock and all genotyped patients, recessive and dominant genetic models. Univariate and multivariate associations are reported. In dominant genetic model in patients with septic shock the SNPs rs2093266 and rs1955656 were significantly associated with KDIGO stage 2–3 AKI after adjustment. (DOC 89 kb)
Additional file 10: Fulfillment of quality criterion by Clark et al. for genetic association studies, which were originally validated for AKI studies by Lu et al., and were later adapted by Vilander et al. Self-assessment of the study quality criteria is shown. (DOC 45 kb)

## Abbreviations

ACCP/SCCM: American College of Chest Physicians/Society of Critical Care Medicine
ACE: Angiotensin-converting enzyme
AKI: Acute kidney injury
AMP: Adenosine monophosphate
ARB: Angiotensin receptor blocker
BCL2: B-cell CLL/lymphoma 2
BMI: Body mass index
COPD: Chronic obstructive pulmonary disease
CRF: Case report form
DNA: Deoxyribonucleic acid
FIMM: Institute for Molecular Medicine Finland
FINNAKI: Finnish Acute Kidney Injury
HWE: Hardy-Weinberg equilibrium
ICU: Intensive care unit
KDIGO: Kidney Disease: Improving Global Outcomes
LD: Linkage disequilibrium
LPS: Lipopolysaccharide
NSAID: Non-steroidal anti-inflammatory drug
OR: Odds ratio
PCI: Protein C inhibitor
SAPS II: Simplified acute physiology score II
SERPINA4: Serpin peptidase inhibitor, clade A (alpha-1 antiproteinase, antitrypsin), member 4
SERPINA5: Serpin peptidase inhibitor, clade A (alpha-1 antiproteinase, antitrypsin), member 5
SIK3: Salt-inducible kinase family 3
SNP: Single nucleotide polymorphism(s)
