# Supplementary material for: Genetic variants in SERPINA4 and SERPINA5, but not BCL2 and SIK3 are associated with acute kidney injury in critically ill patients with septic shock
Source: Crit Care. 2017 Mar 8;21:47. doi: 10.1186/s13054-017-1631-3 (PMC5341446; doi:10.1186/s13054-017-1631-3)
Supplement: Additional file 1: — FINNAKI study enrollment and inclusion of patients. Report of the enrollment period and inclusion and exclusion criteria. (DOC 22 kb) [file 13054_2017_1631_MOESM1_ESM.doc]

Additional file 1. The FINNAKI study enrollment and inclusion of patients.

The patients were enrolled from September 1^st^ 2011 to April 30^th^ 2012. The included patients were adults (>18y) with either 1) emergency intensive care unit (ICU) admission or 2) those postoperatively, electively admitted patients that were expected to stay in the ICU for more than 24 hours. We excluded patients 1) with end-stage renal disease or maintenance dialysis, 2) who were readmitted that previously received renal replacement therapy (RRT), 3) with insufficient language skills or with no permanent residency in Finland, 4) who were transferred between study ICUs if already in the study for 5 days, 5) admitted to intermediate care, and 6) organ donors.
