# Supplementary material for: Genetic variants in SERPINA4 and SERPINA5, but not BCL2 and SIK3 are associated with acute kidney injury in critically ill patients with septic shock
Source: Crit Care. 2017 Mar 8;21:47. doi: 10.1186/s13054-017-1631-3 (PMC5341446; doi:10.1186/s13054-017-1631-3)
Supplement: Additional file 2: — Primer sequences. Polymerase chain reaction (PCR) 1 and 2 and extension primer sequences. (DOC 30 kb) [file 13054_2017_1631_MOESM2_ESM.doc]

Additional file 2. Primer sequences.

| Oligo Name | PCR primer 1 (Sequence 5' to 3') | PCR primer 2 (Sequence 5' to 3') | Extension primer (Sequence 5' to 3') |
| --- | --- | --- | --- |
| rs8094315 | ACGTTGGATGCCTGCCTATGTTTCCTTCAG | ACGTTGGATGTCAGCAGATGGTCAGTTTTC | AGCTTGTCTGGCAAATA |
| rs1955656 | ACGTTGGATGCCGTTTCCCAAGCTTACATC | ACGTTGGATGTCAGAGCTCTTTCTGCATGG | GGGAAAGTCTGAGGTAA |
| rs625145 | ACGTTGGATGAGCTCACTAATAAAACGACC | ACGTTGGATGAGAGGCACATTCAGATCATC | CATTTATGCACTGAACTCA |
| rs12457893 | ACGTTGGATGCTTTGTCAGGCATTGTTTTG | ACGTTGGATGAGAACAGTGGTGAGCTTGTC | GGCATTGTTTTGCATTTTTA |
| rs2093266 | ACGTTGGATGTGGAGGTGAGAAGATGAGAG | ACGTTGGATGTCCCCACTCATGACTTTCAC | TGTTTAACTGATTCTGTTTTCT |
