# Supplementary material for: Genetic variants in SERPINA4 and SERPINA5, but not BCL2 and SIK3 are associated with acute kidney injury in critically ill patients with septic shock
Source: Crit Care. 2017 Mar 8;21:47. doi: 10.1186/s13054-017-1631-3 (PMC5341446; doi:10.1186/s13054-017-1631-3)
Supplement: Additional file 3: — Detailed power calculations for each SNP, for power to report the additionional risk given known minor allele frequencies and risk ratios. (DOC 22 kb) [file 13054_2017_1631_MOESM3_ESM.doc]

Additional file 3. Detailed power calculations regarding each SNP.

The calculations report the addition to the risk by each additional copy of the allele. In these calculations power was 97.1% for SNP rs8094315 (with minor allele frequency, MAF 0.17 and relative risk, RR 0.61 or 1.64), 97.9% for SNP rs12457893 (with MAF 0.42 and RR 0.71 or 1.41), 78.1% for SNP rs2093266 (with MAF 0.07 and RR 0.55 or 1.82), 72.2% for SNP rs1955656 (with MAF 0.07 and RR 0.57 or 1.75) and 99.9% for SNP rs625145 (with MAF 0.26 and RR 0.66 or 1.52).
