# Supplementary material for: Genetic variants in SERPINA4 and SERPINA5, but not BCL2 and SIK3 are associated with acute kidney injury in critically ill patients with septic shock
Source: Crit Care. 2017 Mar 8;21:47. doi: 10.1186/s13054-017-1631-3 (PMC5341446; doi:10.1186/s13054-017-1631-3)
Supplement: Additional file 4: — Demographic and baseline characteristics of the entire cohort according to the presence of acute kidney injury: the main demographic and baseline characteristics of the patients included in the FINNAKI genetic study. (DOC 86 kb) [file 13054_2017_1631_MOESM4_ESM.doc]

Additional file 4. Demographic and baseline characteristics of the entire cohort according to the presence of acute kidney injury.

| Characteristics | Data available | KDIGO 0 (n = 1545) | KDIGO 2 and 3 (n=601) | All Patients (n=2146) | *p* |
| --- | --- | --- | --- | --- | --- |
| Age (years) | 2146 | 62.0 (48.0-72.0) | 65.0 (54.0-74.0) | 62.0 (50.0-72.0) | <0.0001 |
| Gender (male) | 2146 | 957 (61.9%) | 391 (65.1%) | 1348 (62.8%) | 0.196 |
| BMI (kg/m2) | 2126 | 25.7 (23.2-28.7) | 27.5 (24.5-31.3) | 26.1 (23.5-29.4) | <0.0001 |
| Co-morbidities |  |  |  |  |  |
| Arterial hypertension | 2137 | 626 (40.8%) | 319 (53.1%) | 945 (44.2%) | <0.0001 |
| Diabetes | 2144 | 271 (17.6%) | 165 (27.5%) | 436 (20.3%) | <0.0001 |
| Arteriosclerosis | 2130 | 156 (10.2%) | 91 (15.2%) | 247 (11.6%) | 0.002 |
| COPD | 2137 | 135 (8.8%) | 42 (7.0%) | 177 (8.3%) | 0.221 |
| Chronic liver disease | 2122 | 49 (3.2%) | 43 (7.2%) | 92 (4.3%) | <0.0001 |
| Systolic heart failure | 2134 | 136 (8.9%) | 74 (12.4%) | 210 (9.8%) | 0.019 |
| Thromboembolus | 2135 | 86 (5.6%) | 29 (4.8%) | 115 (5.4%) | 0.524 |
| Rehumatic vasculitis | 2130 | 69 (4.5%) | 25 (4.2%) | 94 (4.4%) | 0.815 |
| Serum creatinine |  |  |  |  |  |
| Baseline (μmol/l) | 2144 | 79.0 (67.0-94.0) | 81.0 (68.6-94.0) | 80.0 (68.0-94.0) | 0.317 |
| Maximum (μmol/l) | 2146 | 62.0 (47.0-80.0) | 220.0 (138.0-327.0) | 73.0 (52.0-119.0) | <0.0001 |
| Pre-ICU daily medication |  |  |  |  |  |
| ACE inhibitor or ARB | 2099 | 466 (30.9%) | 252 (42.7%) | 718 (34.2%) | <0.0001 |
| NSAID | 2058 | 116 (7.8%) | 71 (12.3%) | 187 (9.1%) | 0.002 |
| Aspirin | 2109 | 370 (24.4%) | 154 (25.9%) | 524 (24.8%) | 0.502 |
| Diuretic | 2106 | 316 (20.9%) | 177 (29.7%) | 493 (23.4%) | <0.0001 |
| Metformin | 2115 | 160 (10.5%) | 106 (17.8%) | 266 (12.6%) | <0.0001 |
| Statin | 2113 | 388 (25.6%) | 189 (31.7%) | 577 (27.3%) | 0.005 |
| Immunosuppressives | 2118 | 62 (4.1%) | 36 (6.0%) | 98 (4.6%) | 0.065 |
| Corticosteroids | 2122 | 103 (6.8%) | 54 (9.0%) | 157 (7.4%) | 0.080 |
| Warfarin | 2116 | 173 (11.4%) | 103 (17.2%) | 276 (13.0%) | 0.001 |
| Treatments administered 48h before admission |  |  |  |  |  |
| Contrast medium | 2138 | 409 (26.6%) | 115 (19.2% | 524 (24.5%) | <0.0001 |
| Aminoglycoside antibiotics | 2143 | 9 (0.6%) | 8 (1.3%) | 17 (0.8%) | 0.102 |
| Peptidoglycan antibiotics | 2143 | 101 (6.5%) | 45 (7.5%) | 146 (6.8%) | 0.445 |
| ACE inhibitor or ARB | 2114 | 321 (21.0%) | 159 (27.0%) | 480 (22.7%) | 0.004 |
| NSAID | 2051 | 138 (9.3%) | 59 (10.4%) | 197 (9.6%) | 0.503 |
| Amfoterisin B | 2141 | 2 (0.1%) | 3 (0.5%) | 5 (0.2%) | 0.137 |
| Diuretics | 2099 | 353 (23.4%) | 210 (35.7%) | 563 (26.8%) | <0.0001 |
| Colloids (gelatin or starch) | 2017 | 386 (26.8%) | 222 (38.3%) | 608 (30.1%) | <0.0001 |
| Albumin | 2113 | 14 (0.9%) | 14 (2.4%) | 28 (1.3%) | 0.018 |
| Admission |  |  |  |  |  |
| Emergency | 2125 | 1354 (88.6%) | 553 (92.6%) | 1907 (89.7%) | 0.005 |
| Operative | 2145 | 542 (35.1%) | 167 (27.8%) | 709 (33.1%) | 0.001 |
| SAPS II score 24h without renal and age components | 2117 | 20.0 (13.0-29.0) | 24.0 (16.0-34.0) | 21.0 (14.0-30.0) | <0.0001 |
| Mechanical ventilation | 2146 | 1006 (65.1%) | 415 (69.1%) | 1421 (66.2%) | 0.084 |
| White blood cell count, maximum (10^9^/L) | 1791 | 11.0 (7.8-15.3) | 12.0 (8.3-17.4) | 11.3 (8.0-15.9) | 0.001 |
| Platelet count, minimum (10^9^/L) | 1974 | 205.0 (153.0-268.5) | 193.0 (118.5-264.0) | 202.0 (143.0-267.0) | <0.0001 |
| Source of infection (percent of all infections) | 2146 |  |  |  | <0.0001 |
| Lung |  | 181 (11.7%) | 100 (16.6%) | 218 (13.1%) |  |
| Abdomen |  | 62 (4.0%) | 78 (13.0%) | 140 (6.5%) |  |
| Urinary tract |  | 10 (0.6%) | 26 (4.3%) | 36 (1.7%) |  |
| Skin |  | 25 (1.6%) | 26 (4.3%) | 51 (2.4%) |  |
| Others |  | 19 (1.2%) | 8 (1.5%) | 28 (1.3%) |  |
| Multiple sources |  | 21 (1.4%) | 16 (2.7%) | 37 (1.7%) |  |
| Unknown source of infection |  | 1227 (79.4%) | 346 (57.6%) | 1573 (73.3%) |  |
| Values given for variables: for continuous variables median (interquartile range), for categorical variables total number (percent of affected in a group).  Statistics used for significance: for continuous variables Independent Samples Mann Whitney U Test, for categorical variables Fischer's Exact Test. | | | | | |
