# Supplementary material for: Genetic variants in SERPINA4 and SERPINA5, but not BCL2 and SIK3 are associated with acute kidney injury in critically ill patients with septic shock
Source: Crit Care. 2017 Mar 8;21:47. doi: 10.1186/s13054-017-1631-3 (PMC5341446; doi:10.1186/s13054-017-1631-3)
Supplement: Additional file 5: — Logistic regression (the “enter” method) in patients with sepsis (n = 653) in differing demographic variables, missing data imputed. Number (N) of imputed values and percentages of the cohort are given. Results of logistic regression in patients with sepsis showing that higher BMI, not having COPD, use of NSAID as daily medication, administration of contrast medium prior to ICU admission, SAPS II score without renal or age components, and source of infection were significantly associated with KDIGO stage 2–3 AKI. (DOC 54 kb) [file 13054_2017_1631_MOESM5_ESM.doc]

Additional file 5. Logistic regression (Enter method) in patients with sepsis (n=653) in differing demographic variables, missing data imputed. Number (N) of imputed values given, as well as percentage of the cohort.

| Characteristic | Odds Ratio (95% Confidence Interval) | *p* | Imputed values (%) |
| --- | --- | --- | --- |
| Age | 1.01 (1.00-1.02) | 0.13 | 0 |
| Gender (male) | 0.89 (0.62-1.28) | 0.54 | 0 |
| BMI | 1.04 (1.01-1.07) | 0.011 | 2(0.31) |
| Arterial hypertension | 0.94 (0.60-1.47) | 0.78 | 2(0.31) |
| Diabetes | 1.26 (0.82-1.95) | 0.30 | 0 |
| Arteriosclerosis | 1.63 (0.94-1.85) | 0.083 | 5(0.77) |
| COPD | 0.35 (0.19-0.66) | 0.001 | 4(0.61) |
| Chronic liver disease | 1.80 (0.87-3.71) | 0.12 | 6(0.92) |
| Pre-ICU daily ACE inhibitor or ARB | 1.03 (1.66-1.62) | 0.90 | 13(2.0) |
| Pre-ICU daily NSAID | 1.96 (1.15-3.34) | 0.013 | 29(4.4) |
| Contrast medium within 48h before admission | 0.49 (0.31-0.77) | 0.002 | 1(0.15) |
| Diuretics within 48h before admission | 1.07 (0.73-1.57) | 0.71 | 16(2.5) |
| Colloids within 48h before admission | 1.29 (0.89-1.88) | 0.18 | 33(5.1) |
| Platelet count, minimum | 1.00 (1.00-1.00) | 0.099 | 21(3.2) |
| SAPS II score 24h wo renal and age points | 1.03 (1.02-1.05) | <0.0001 | 4(0.61) |
| Mechanical ventilation | 1.45 (0.95-2.21) | 0.085 | 0 |
| Source of infection |  | <0.0001 | 0 |
| Lungs (reference) | 1.00 |  |  |
| Abdomen | 2.51 (1.56-4.05) | <0.0001 |  |
| Urinary tract | 5.09 (2.15-12.04) | <0.0001 |  |
| Skin | 1.82 (0.95-3.50) | 0.073 |  |
| Others | 0.79 (0.32-1.92) | 0.60 |  |
| Several | 1.59 (0.75-3.37) | 0.23 |  |
| Unknown | 2.30 (1.33-3.99) | 0.003 |  |
|  |  |  |  |
| Abbreviations: ACE, Angiotensin-Converting Enzyme; ARB, Angiotensin Receptor Blocker; BMI, Body Mass Index; COPD, Chronic Obstructive Pulmonary Disease; ICU, Intensive Care Unit; NSAID, Non-steroidal Anti-Inflammatory Drug; SAPS II, Simplified Acute Physiology Score II. | | |  |
| The Hosmer and Lemeshow Test gave value 6.718 and remained insignificant, *p*=0.567. | | |  |
