# Supplementary material for: Genetic variants in SERPINA4 and SERPINA5, but not BCL2 and SIK3 are associated with acute kidney injury in critically ill patients with septic shock
Source: Crit Care. 2017 Mar 8;21:47. doi: 10.1186/s13054-017-1631-3 (PMC5341446; doi:10.1186/s13054-017-1631-3)
Supplement: Additional file 7: — Association between acute kidney injury and the polymorphisms studied in all genotyped patients (n = 2146) (additive genetic model). Association between acute kidney injury and the studied polymorphisms in all genotyped patients was tested in univariate and multivariate models. No significant associations are reported. (DOC 35 kb) [file 13054_2017_1631_MOESM7_ESM.doc]

Additional file 7. Association between acute kidney injury and the studied polymorphisms in all genotyped patients (n=2146). Additive genetic model.

| Single Nucleotide Polymorphism | Chromo-some | Base-pair Position | Gene and alleles (Major/minor) | Minor Allele Frequency^a^ | Univariate Odds Ratio | Univariate *p* | Multivariate Odds Ratio | Multivariate *p* |
| --- | --- | --- | --- | --- | --- | --- | --- | --- |
|
| rs625145 | 11 | 116857220 | *SIK3* A/T | 0.21/0.20 | 1.05 | 0.55 | 1.08 | 0.42 |
| rs1955656 | 14 | 94579038 | *SERPINA5* G/A | 0.10/0.10 | 0.97 | 0.76 | 0.93 | 0.58 |
| rs2093266 | 14 | 94566450 | *SERPINA4* G/A | 0.10/0.10 | 0.97 | 0.76 | 0.93 | 0.58 |
| rs8094315 | 18 | 63268814 | *BCL2* A/G | 0.23/0.24 | 0.92 | 0.33 | 0.92 | 0.37 |
| rs12457893 | 18 | 63258928 | *BCL2* A/C | 0.37/0.38 | 0.96 | 0.59 | 0.99 | 0.89 |
| Abbreviations: *BCL2*, B-cell CLL/lymphoma 2–gene; *SERPINA4*, serpin peptidase inhibitor, clade A (alpha-1 antiproteinase, antitrypsin), member 4 –gene; *SERPINA5*, serpin peptidase inhibitor, clade A (alpha-1 antiproteinase, antitrypsin), member 5 –gene; *SIK3*, salt-inducible kinase family 3 –gene. | | | | | | | | |
| ^a^patients/controls | | | | | | | | |
