# Supplementary material for: Genetic variants in SERPINA4 and SERPINA5, but not BCL2 and SIK3 are associated with acute kidney injury in critically ill patients with septic shock
Source: Crit Care. 2017 Mar 8;21:47. doi: 10.1186/s13054-017-1631-3 (PMC5341446; doi:10.1186/s13054-017-1631-3)
Supplement: Additional file 8: — Logistic regression (“enter” method) in all genotyped patients (n = 2146) in differing demographic variables, missing data imputed. Number (N) of imputed values and the percentages of the cohort are given. Logistic regression in all genotyped patients showed that BMI, chronic liver disease, use of NSAID as daily medication, use of warfarin as daily medication, administration of contrast medium prior to ICU admission, administration of colloids prior to ICU admission, administration of albumin prior to ICU admission, maximum white blood cell count, minimum platelet count, SAPS II without age or renal components, operative admission, and source of infection were significantly associated with KDIGO stage 2–3 AKI. (DOC 72 kb) [file 13054_2017_1631_MOESM8_ESM.doc]

Additional file 8. Logistic regression (Enter method) in all genotyped patients (n=2146) in differing demographic variables, missing data imputed. Number (N) of imputed values given, as well as percentage of the cohort.

| Characteristic | Odds Ratio (95% Confidence Interval) | *p* | Imputed values (%) |
| --- | --- | --- | --- |
| Age | 1.01 (1.00-1.01) | 0.085 | 0 |
| Gender (male) | 1.07 (0.86-1.34) | 0.52 | 0 |
| BMI | 1.05 (1.03-1.07) | <0.0001 | 20(0.93) |
| Arterial hypertension | 1.07 (0.81-1.41) | 0.62 | 9(0.42) |
| Diabetes | 1.35 (0.93-1.95) | 0.11 | 2(0.093) |
| Arteriosclerosis | 1.33 (0.96-1.85) | 0.084 | 16(0.75) |
| Chronic liver disease | 2.14 (1.33-3.44) | 0.002 | 24(1.1) |
| Systolic cardiac failure | 0.97 (0.67-1.41) | 0.87 | 12(0.56) |
| Pre-ICU daily ACE inhibitor or ARB | 1.41 (1.00-1.99) | 0.051 | 47(2.2) |
| Pre-ICU daily NSAID | 1.51 (1.07-2.13) | 0.019 | 88(4.1) |
| Pre-ICU daily diuretic | 0.83 (0.61-1.12) | 0.24 | 40(1.9) |
| Pre-ICU daily metformin | 1.02 (0.66-1.58) | 0.92 | 31(1.4) |
| Pre-ICU daily statin | 1.01 (0.77-1.32) | 0.95 | 33(1.5) |
| Pre-ICU daily immunosuppressives | 1.08 (0.64-1.82) | 0.77 | 28(1.3) |
| Pre-ICU daily corticosteroids | 0.94 (0.62-1.44) | 0.79 | 24(1.1) |
| Pre-ICU daily warfarin | 1.41 (1.02-1.94) | 0.040 | 30(1.4) |
| Contrast medium within 48h before admission | 0.62 (0.48-0.81) | <0.0001 | 8(0.37) |
| Aminoglycosides within 48h before admission | 1.77 (0.60-5.25) | 0.30 | 3(0.14) |
| ACE inhibitor or ARB within 48h before admission | 0.74 (0.53-1.03) | 0.075 | 32(1.5) |
| Amfoterisin within 48h before admission | 3.50 (0.41-30.08) | 0.25 | 5(0.23) |
| Diuretics within 48h before admission | 1.18 (0.90-1.55) | 0.24 | 47(2.2) |
| Colloids within 48h before admission | 1.78 (1.39-2.28) | <0.0001 | 129(6.0) |
| Albumin within 48h before admission | 2.75 (1.15-6.57) | 0.023 | 33(1.5) |
| White blood cell count, maximum | 1.03 (1.01-1.04) | 0.001 | 355(16.5) |
| Platelet count, minimum | 1.00 (1.00-1.00) | 0.001 | 172(8.0) |
| Mechanical ventilation | 1.09 (0.83-1.44) | 0.52 | 0 |
| SAPS II score 24h wo renal and age points | 1.02 (1.01-1.03) | 0.001 | 29(1.4) |
| Operative admission | 0.60 (0.45-0.80) | 0.001 | 1(0.047) |
| Emergency admission | 1.15 (0.74-1.79) | 0.53 | 21(0.98) |
| Source of infection |  | <0.0001 | 0 |
| Lungs | 1.00 |  |  |
| Abdomen | 2.69 (1.68-4.32) | <0.0001 |  |
| Urinary tract | 3.37 (1.48-7.68) | 0.004 |  |
| Skin | 1.77 (0.93-3.38) | 0.082 |  |
| Others | 0.98 (0.40-2.39) | 0.97 |  |
| Several | 1.55 (0.74-3.26) | 0.25 |  |
| Unknown | 0.68 (0.50-0.92) | 0.012 |  |
| Abbreviations: ACE, Angiotensin-Converting Enzyme; ARB, Angiotensin Receptor Blocker; BMI, Body Mass Index; COPD, Chronic Obstructive Pulmonary Disease; ICU, Intensive Care Unit; NSAID, Non-steroidal Anti-Inflammatory Drug; SAPS II, Simplified Acute Physiology Score II.  The Hosmer and Lemeshow test gave Chi-square of 5.457 and *p*-value remained insignificant, 0.708. | | |  |
