# Supplementary material for: Genetic variants in SERPINA4 and SERPINA5, but not BCL2 and SIK3 are associated with acute kidney injury in critically ill patients with septic shock
Source: Crit Care. 2017 Mar 8;21:47. doi: 10.1186/s13054-017-1631-3 (PMC5341446; doi:10.1186/s13054-017-1631-3)
Supplement: Additional file 9: — Association between acute kidney injury and the studied polymorphisms, recessive and dominant genetic models. Association between acute kidney injury and the polymorphisms studied in septic patients, patients with septic shock and all genotyped patients, recessive and dominant genetic models. Univariate and multivariate associations are reported. In dominant genetic model in patients with septic shock the SNPs rs2093266 and rs1955656 were significantly associated with KDIGO stage 2–3 AKI after adjustment. (DOC 89 kb) [file 13054_2017_1631_MOESM9_ESM.doc]

Additional file 9. Association between acute kidney injury and the studied polymorphisms, recessive and dominant genetic models.

| Septic patients |  |  |  |  |  |  |
| --- | --- | --- | --- | --- | --- | --- |
| SNP | Gene and alleles (Major/minor) | Genotype | Univariate Odds Ratio | Univariate *p* | Multivariate Odds Ratio | Multivariate *p* |
|  |  | available |  |  |  |  |
| recessive |  |  |  |  |  |  |
| rs625145 | *SIK3* A/T | 653 | 0.81 | 0.64 | 0.87 | 0.77 |
| rs1955656 | *SERPINA5* G/A | 653 | 1.19 | 0.84 | 0.78 | 0.77 |
| rs2093266 | *SERPINA4* G/A | 653 | 1.19 | 0.84 | 0.78 | 0.77 |
| rs8094315 | *BCL2* A/G | 650 | 1.13 | 0.74 | 0.93 | 0.85 |
| rs12457893 | *BCL2* A/C | 653 | 0.87 | 0.55 | 0.79 | 0.37 |
| dominant |  |  |  |  |  |  |
| rs625145 | *SIK3* A/T | 653 | 0.87 | 0.41 | 0.92 | 0.64 |
| rs1955656 | *SERPINA5* G/A | 653 | 0.73 | 0.11 | 0.72 | 0.13 |
| rs2093266 | *SERPINA4* G/A | 653 | 0.73 | 0.11 | 0.72 | 0.13 |
| rs8094315 | *BCL2* A/G | 650 | 1.12 | 0.48 | 1.13 | 0.48 |
| rs12457893 | *BCL2* A/C | 653 | 1.11 | 0.53 | 1.24 | 0.24 |
| Patients with septic shock |  |  |  |  |  |  |
| SNP | Gene and alleles (Major/minor) | Genotype | Univariate Odds Radio | Univariate *p* | Multivariate Odds Ratio | Multivariate *p* |
|  |  | available |  |  |  |  |
| recessive |  |  |  |  |  |  |
| rs625145 | *SIK3* A/T | 478 | 1.01 | 0.99 | 1.00 | 1.00 |
| rs1955656 | *SERPINA5* G/A | 478 | 1.35 | 0.74 | 0.77 | 0.79 |
| rs2093266 | *SERPINA4* G/A | 478 | 1.35 | 0.74 | 0.77 | 0.79 |
| rs8094315 | *BCL2* A/G | 476 | 1.43 | 0.41 | 0.95 | 0.92 |
| rs12457893 | *BCL2* A/C | 478 | 1.21 | 0.50 | 1.00 | 1.00 |
| dominant |  |  |  |  |  |  |
| rs625145 | *SIK3* A/T | 478 | 0.75 | 0.13 | 0.74 | 0.14 |
| rs1955656 | *SERPINA5* G/A | 478 | 0.65 | 0.065 | 0.59 | 0.034 |
| rs2093266 | *SERPINA4* G/A | 478 | 0.65 | 0.065 | 0.59 | 0.034 |
| rs8094315 | *BCL2* A/G | 476 | 1.20 | 0.33 | 1.20 | 0.37 |
| rs12457893 | *BCL2* A/C | 478 | 1.36 | 0.11 | 1.45 | 0.072 |
| All patients |  |  |  |  |  |  |
| SNP | Gene and alleles (Major/minor) | Genotype | Univariate Odds Ratio | Univariate *p* | Multivariate Odds Ratio | Multivariate *p* |
|  |  | available |  |  |  |  |
| recessive |  |  |  |  |  |  |
| rs625145 | *SIK3* A/T | 2146 | 1.17 | 0.50 | 1.10 | 0.71 |
| rs1955656 | *SERPINA5* G/A | 2146 | 1.64 | 0.31 | 1.11 | 0.85 |
| rs2093266 | *SERPINA4* G/A | 2146 | 1.64 | 0.31 | 1.11 | 0.85 |
| rs8094315 | *BCL2* A/G | 2141 | 0.84 | 0.43 | 0.78 | 0.30 |
| rs12457893 | *BCL2* A/C | 2146 | 0.89 | 0.40 | 0.91 | 0.53 |
| dominant |  |  |  |  |  |  |
| rs625145 | *SIK3* A/T | 2146 | 1.04 | 0.67 | 1.09 | 0.42 |
| rs1955656 | *SERPINA5* G/A | 2146 | 0.93 | 0.58 | 0.92 | 0.52 |
| rs2093266 | *SERPINA4* G/A | 2146 | 0.93 | 0.58 | 0.92 | 0.52 |
| rs8094315 | *BCL2* A/G | 2141 | 0.92 | 0.42 | 0.94 | 0.54 |
| rs12457893 | *BCL2* A/C | 2146 | 0.99 | 0.88 | 1.03 | 0.80 |
|  |  |  |  |  |  |  |
| Abbreviations: *BCL2*, B-cell CLL/lymphoma 2–gene; *SERPINA4*, serpin peptidase inhibitor, clade A (alpha-1 antiproteinase, antitrypsin), member 4 –gene; *SERPINA5*, serpin peptidase inhibitor, clade A (alpha-1 antiproteinase, antitrypsin), member 5 –gene; *SIK3*, salt-inducible kinase family 3 –gene; SNP, Single Nucleotide Polymorphism. | | | | | | |
| ^a^patients/controls | | | | | | |
