# Supplementary material for: Genetic variants in SERPINA4 and SERPINA5, but not BCL2 and SIK3 are associated with acute kidney injury in critically ill patients with septic shock
Source: Crit Care. 2017 Mar 8;21:47. doi: 10.1186/s13054-017-1631-3 (PMC5341446; doi:10.1186/s13054-017-1631-3)
Supplement: Additional file 10: — Fulfillment of quality criterion by Clark et al. for genetic association studies, which were originally validated for AKI studies by Lu et al., and were later adapted by Vilander et al. Self-assessment of the study quality criteria is shown. (DOC 45 kb) [file 13054_2017_1631_MOESM10_ESM.doc]

Additional file 10. Fulfillment of quality criterion by Clark et al [1] for genetic association studies, originally validated for AKI studies by Lu et al [2], later adapted by Vilander et al [3].

| Criterion | Criteria used | Fulfills the criterion | Page, row |
| --- | --- | --- | --- |
| 1. Defining the Control Group | Control group described for size in relation to case group (n > case group) and ethnicity, in sufficient detail to allow replication. Cohort studies are given a point provided that the cohort is described in a sufficient detail, namely the ethnicity of a cohort is given. Prospective cohorts regarded as strong design in demonstrating causality between genetic polymorphism and the development of AKI. Size of the control group sufficient when essentially equivalent with case group. | Yes. | Page 4, row 80. Page 7, row 161. |
| 2. Hardy-Weinberg Equilibrium | Case and control groups assessed for Hardy-Weinberg equilibrium (HWE). Cohort or control group should be in HWE (p>0.01). In the case of multiple polymorphisms, majority of them should be in HWE. | Yes. | Page 8, row 182. |
| 3. Defining the Case Group | The disease state of interest as well as inclusion and exclusion criteria of the case group defined in sufficient detail to allow replication. Studies that aim to investigate predisposition to AKI are given a point only if they describe the phenotype. | Yes. | Page 4, row 80.  Page 5, row 101. |
| 4. Primer Sequence | The priming sequences used for genotyping or references to them provided. Whenever a commercial assay was used for genotyping, a point was given as well. | Yes. | Page 5, row 121. |
| 5. Describing the Methods | Genotyping method described in sufficient detail to allow replication. In addition, second validating assay performed or the accuracy of the assay validated. Accepted quality control methods: duplicated samples or control samples included, second independent investigator analyzing the genotype calls, repeating of ambiguous results. | Yes. | Page 5, row 117. |
| 6. Blinding | Genotyping performed blinded to clinical status. | Yes. | Page 6, row 129. |
| 7. Power Calculation | Prospective or retrospective power calculation performed. | Yes. | Page 7, row 151. |
| 8. Statistics | Tests of significance such as p values, confidence intervals or odds ratios presented. | Yes. | Page 8, row 183. |
| 9. Corrected Statistics | Corrected for the increased risk of false-positive error in the case of multiple polymorphisms studied. Should X2 or odds ratio be used, Hardy-Weinberg equilibrium is to be assessed. Where there was only one polymorphism studied, a point was given. | No. |  |
| 10. Replication | Study performing second, confirmatory study or confirming earlier polymorphism study. A point was given only for studies performing exact replication of a polymorphism in association with a phenotype. | Yes. | Page 4, row 74. |

References

1. Clark MF, Baudouin SV: **A systematic review of the quality of genetic association studies in human sepsis.** *Intensive Care Med* 2006, **32**(11):1706-1712.

2. Lu JC. Coca SG. Patel UD. Cantley L. Parikh CR. Translational Research Investigating Biomarkers and Endpoints for Acute Kidney Injury (TRIBE-AKI) Consortium: **Searching for genes that matter in acute kidney injury: a systematic review.** *Clin J Am Soc Nephrol* 2009, **4**(6):1020-1031.

3. Vilander LM, Kaunisto MA, Pettilä V: **Genetic predisposition to acute kidney injury - a systematic review.** *BMC Nephrology* 2015, **16**(1):1-10.
